# Brexpiprazole Treatment in a Child With Autism Spectrum Disorder, Attention-Deficit/Hyperactivity Disorder (ADHD), and Trauma-Associated Hoarding Symptoms: A Case Report

**DOI:** 10.7759/cureus.110553

**Published:** 2026-06-09

**Authors:** Makiko Ikeda, Manabu Takaki

**Affiliations:** 1 Department of Psychosocial Medicine, National Center for Child Health and Development, Tokyo, JPN; 2 Department of Neuropsychiatry, Graduate School of Medicine, Dentistry, and Pharmaceutical Sciences, Okayama University, Okayama, JPN

**Keywords:** attention-deficit/hyperactivity disorder, autism spectrum disorder, brexpiprazole, hoarding disorder, posttraumatic stress disorder (ptsd)

## Abstract

Children with autism spectrum disorder (ASD) and attention-deficit/hyperactivity disorder (ADHD) frequently present with multiple psychiatric comorbidities, often accompanied by severe emotional dysregulation and behavioral disturbances that complicate diagnosis and treatment. We describe a child with comorbid ASD and ADHD who also met diagnostic criteria for hoarding disorder and separation anxiety disorder and exhibited trauma-related symptoms. The patient showed minimal response to psychotherapy and conventional pharmacological interventions. Although comprehensive multidisciplinary support was provided, severe emotional outbursts and irritability persisted, and treatment with approved antipsychotics and ADHD medications was limited by adverse effects. Adjunctive treatment with brexpiprazole was subsequently initiated. Following treatment, some improvement in hoarding behaviors and social motivation was observed.

This case highlights the therapeutic challenges associated with managing complex neurodevelopmental conditions with multiple psychiatric comorbidities. These findings may provide insight into the interaction between neurodevelopmental vulnerability and trauma-related psychopathology and suggest a possible pharmacological consideration for treatment-resistant cases.

## Introduction

Autism spectrum disorder (ASD) frequently co-occurs with attention-deficit/hyperactivity disorder (ADHD), with more than half of children with ASD meeting diagnostic criteria for ADHD [1]. The coexistence of these conditions poses substantial diagnostic and therapeutic challenges because of overlapping cognitive deficits, communication difficulties, and reduced adaptability to environmental demands [2]. Moreover, individuals with comorbid ASD and ADHD exhibit higher rates of psychiatric comorbidities and behavioral problems than those with either condition alone [3].

Hoarding disorder (HD) has been reported in association with both ASD and ADHD. HD is characterized by persistent difficulty discarding possessions regardless of their actual value, resulting in excessive accumulation that compromises daily functioning [4]. Restricted interests and insistence on sameness in ASD may contribute to hoarding behaviors, which have been reported more frequently in children with ASD than in typically developing peers [5]. Clinically significant hoarding behaviors have also been observed in young individuals with ADHD, particularly in those with greater inattention and behavioral dysregulation [6]. These findings suggest shared neurocognitive mechanisms among ASD, ADHD, and HD, including executive dysfunction and impaired decision-making [2].

Anxiety and trauma-related psychopathology may further interact with the neurocognitive vulnerabilities of ASD and ADHD, contributing to more severe and persistent hoarding symptoms. Individuals with ASD and ADHD are known to exhibit elevated anxiety levels [2,5] and increased vulnerability to trauma-related symptoms [7]. In such cases, patients may present with a more severe and clinically complex phenotype, making treatment particularly challenging.

Although antipsychotics may improve certain behavioral symptoms associated with ASD, treatment response and tolerability vary considerably across individuals. ADHD medications often show reduced efficacy and poorer tolerability in individuals with comorbid ASD [2], and SSRIs commonly used for HD may be difficult to use in children with prominent self-injurious and aggressive behaviors. In children and adolescents, treatment strategies should also consider developmental immaturity in emotional regulation as well as irritability and hyperarousal associated with trauma-related symptoms [7]. Therefore, therapeutic approaches are needed that can reduce impulsivity and irritability without exacerbating anxiety or emotional overactivation.

From this perspective, brexpiprazole may represent a potentially useful treatment option because of its favorable tolerability profile and possible anxiolytic effects [8,9]. Here, we describe the clinical course of a difficult-to-treat case of HD associated with comorbid ASD, ADHD, anxiety symptoms, and trauma-related symptoms in which brexpiprazole was administered during clinical management.

## Case presentation

A three-year-old male child initially presented with delayed communication development and difficulty participating in group activities. Clinical evaluation revealed impaired reciprocal social interaction, difficulty developing age-appropriate peer relationships, restricted and repetitive behaviors characterized by strong preoccupation with specific toy characters and insistence on routines, and marked sensory sensitivities, including food selectivity and tactile hypersensitivity. He was diagnosed with ASD according to the Diagnostic and Statistical Manual of Mental Disorders, Fifth Edition (DSM-5) criteria. Subsequently, the patient developed hyperactivity, excessive talkativeness, impaired impulse control, intense distress during separation from the mother, excessive worry about separation from the mother, and refusal to go outside because of fear of separation. Based on DSM-5 criteria, he was additionally diagnosed with ADHD and separation anxiety disorder.

The patient participated in a community-based group therapy program, and psychoeducation regarding behavioral management was provided to both parents. The mother experienced depressive symptoms related to caregiving burden and received psychiatric treatment. The father appeared to exhibit similar trait characteristics and maintained an extensive figurine collection, although he was largely unreceptive to intervention.

With individualized support, the patient attended kindergarten; however, beginning in the first grade, he experienced persistent psychosocial difficulties, particularly related to peer teasing and conflict with school staff. During this period, pronounced hoarding behaviors emerged. He accumulated various objects, including leaves, stones, magazines, discarded toys, and empty food containers, and experienced marked distress when asked to discard them. The resulting clutter caused significant family conflict and functional impairment. These symptoms fulfilled DSM-5 diagnostic criteria for HD and were considered distinct from ASD-related restricted interests.

In addition to the hoarding symptoms, engagement with preferred characters appeared to function as a coping strategy for distress related to adverse school experiences. By the third grade, despite placement in a special education resource room, he exhibited self-injurious behavior, verbal and physical aggression, intrusive memories, avoidance, hypervigilance, irritability, and sleep disturbance related to repeated aversive school experiences. Although the patient exhibited trauma-related symptoms consistent with DSM-5 posttraumatic stress disorder (PTSD) criteria other than Criterion A, a formal diagnosis of PTSD was not made.

Despite repeated collaboration with the school, it remained difficult to establish a sufficiently consistent management approach, and some attempts at accommodation may have unintentionally contributed to increased emotional dysregulation. Attempts to promote introspection or address behavioral problems were consistently rejected. Eventually, the patient discontinued school attendance in the fifth grade, after which the severity of anger and emotional dysregulation appeared to decrease slightly, possibly reflecting increased psychological safety outside the school environment. At around the same time, home-based support through visiting nursing services was initiated. Although the patient remained largely unreceptive to advice or behavioral guidance, opportunities were established to engage with him through conversations about his preferred characters.

Risperidone was initiated at 8.5 years of age and titrated to 1 mg/day, although improvement in emotional regulation was limited. Valproic acid was subsequently introduced and increased to 1,200 mg/day (serum concentration: 63.5 μg/mL), together with three traditional Chinese herbal medicines. Although aggressive behavior partially improved, hoarding behaviors and aggression persisted. Trials of methylphenidate, guanfacine, and atomoxetine were discontinued because of adverse effects, including tic exacerbation and worsening anger dysregulation.

Risperidone was discontinued after approximately three years because of excessive weight gain (15-kg increase; obesity degree +73% at age 12 years) and concerns regarding the risk of type 2 diabetes mellitus, after which irritability and aggression worsened. Selective serotonin reuptake inhibitors were avoided because of concerns regarding self-injurious and aggressive behaviors. A trial of zotepine moderately improved anger dysregulation but caused marked fatigue, leading to discontinuation. Aripiprazole further exacerbated irritability.

After obtaining the patient’s assent and written parental consent for off-label treatment, brexpiprazole was initiated at 1 mg/day. Two weeks later, the patient began spontaneously referring to his preferred character while accepting disposal of previously hoarded objects. He also independently created an “outing ticket,” which allowed his mother to leave the home without accompaniment. Three months after initiation of brexpiprazole, hyperactivity and emotional outbursts persisted; however, he spoke less frequently about distressing school experiences. Participation in an online school environment also facilitated gradual improvement in social interaction despite persistent irritability.

Clinical assessments

At 10.3 years of age, the patient was evaluated using the Wechsler Intelligence Scale for Children-Fourth Edition (WISC-IV) [10]. The Full-Scale IQ was 70, with index scores of 97 for the Verbal Comprehension Index, 71 for the Perceptual Reasoning Index, 65 for the Working Memory Index, and 61 for the Processing Speed Index.

Clinical symptoms were assessed at baseline (T0) and one month after initiation of brexpiprazole (T1). Standardized measures included the Social Responsiveness Scale, Second Edition (SRS-2) [11], Japanese version of the Child Behavior Checklist (CBCL/4-18) [12], ADHD Rating Scale-IV (ADHD-RS-IV) [13], and the Children’s Saving Inventory (CSI) [14]. The WISC-IV, SRS-2, and CBCL/4-18 were administered in accordance with the permissions and guidelines of the respective copyright holders. ADHD-RS-IV was administered as part of routine clinical assessment.

Because no validated Japanese version of the CSI was available, a translated version was used with permission from the original author. Results are summarized in Table 1. The SRS-2 total T-score showed a modest reduction from T0 to T1, with the most notable improvement observed in the Social Motivation subscale, which decreased from 70 to 53. Mild changes were also observed in the Social Awareness and Social Cognition domains, whereas the Communication domain remained largely unchanged, and the Restricted and Repetitive Behavior subscale worsened over time.

**Table 1:** Changes in psychological measures before and after brexpiprazole treatment T0: immediately prior to treatment initiation; T1: one month after initiation of brexpiprazole A T-score is a standardized score with a mean of 50 and a standard deviation of 10, used to compare an individual’s score to a normative sample ADHD, Attention-Deficit/Hyperactivity Disorder Rating Scale; CBCL, Child Behavior Checklist; CGI-S, Clinical Global Impressions-Severity of Illness Scale; CSI, Child Saving Inventory; SRS-2: Social Responsiveness Scale, Second Edition

Standardized neurological assessment		t0	t1
SRS-2 (T score)	Total score	74	68
	Social awareness	63	53
	Social cognition	60	50
	Social communication	71	71
	Social motivation	70	53
	Restricted and repetitive behaviors	85	89
CBCL/6-18 (T score)	Total score	79	80
	Internalizing scale	67	67
	Externalizing scale	76	77
	Anxious/depressed	70	70
	Withdrawn/depressed	63	63
	Somatic complaints	50	50
	Social problem	74	70
	Thought problems	78	78
	Attention problems	72	74
	Rule-breaking behavior	70	70
	Aggressive behavior	77	78
ADHD-RS-Ⅳ	Total score	20	20
	Hyperactivity/impulsivity	15	15
	Inattention	5	5
CSI	Total score	87	62
	Discarding	27	16
	Clutter	27	21
	Acquisition	20	14
	Distress/impairment	13	11
CGI-S	-	7	6

The total CSI score decreased substantially from 87 at T0 to 62 at T1. Improvements were observed across all four CSI subscales, with the largest reduction identified in the Discarding domain. Improvements in the Clinical Global Impression scores also suggested overall clinical stabilization.

In contrast, the CBCL showed no meaningful changes in either externalizing or internalizing problems. Only a slight improvement was observed in the Social Problems domain, whereas aggressive behavior and rule-breaking behavior remained unchanged. Similarly, no significant improvement was detected on the ADHD-RS-IV. Because the patient exhibited marked resistance to psychiatric assessment, a comprehensive assessment of trauma-related psychopathology was not feasible.

## Discussion

The present case represented a highly complex clinical phenotype characterized by comorbid ASD, ADHD, anxiety symptoms, trauma-related symptoms, and HD, highlighting the challenges associated with diagnosing and treating co-occurring psychiatric conditions in individuals with neurodevelopmental disorders [2]. Trauma-related psychopathology in individuals with ASD or ADHD may exacerbate irritability, impulsivity, sleep disturbance, and externalizing behaviors. In autistic individuals, experiences such as chronic bullying, social exclusion, interpersonal conflict, and sensory overwhelm may be experienced as highly distressing even when they do not meet DSM-5 Criterion A for PTSD [4]. Moreover, trauma-related symptoms in autistic individuals may present atypically, including emotional outbursts, aggression, withdrawal, worsening repetitive behaviors, and self-injurious behavior, many of which were characteristic features of the present case [7].

In addition, cognitive vulnerabilities observed in this case, including attentional and working memory deficits suggested by the WISC profile, as well as limited verbalization of emotional experiences, may have contributed to the persistence and severity of trauma-related symptoms [7]. These neurocognitive and emotional vulnerabilities may also have complicated the patient’s ability to process and regulate emotional distress.

The clinical course also provided insight into the relationship between hoarding symptoms and trauma-related psychopathology. Although the patient initially showed preoccupation with specific objects consistent with ASD-related restricted interests, worsening trauma-related anxiety and emotional dysregulation were associated with broader and more severe hoarding behaviors, including difficulty discarding ordinary items such as food containers. Storch et al. [5] reported that difficulty discarding is primarily associated with anxiety-related processes, whereas excessive acquiring behaviors are more closely related to externalizing symptoms and attentional dysfunction. In the present case, the most marked improvement following treatment was observed in the “Discarding” domain of the CSI. These findings suggest that ASD-related perseverative tendencies may have been exacerbated by trauma-associated emotional dysregulation, resulting in a more severe hoarding phenotype.

Brexpiprazole has high affinity for α1B- and α2C-adrenergic receptors and acts as an antagonist at these sites, a pharmacological profile associated with stress-modulating and anxiolytic effects in preclinical studies [8]. In the present case, aripiprazole, which is approved in Japan for irritability associated with ASD, exacerbated irritability. By contrast, brexpiprazole was considered a more suitable option because its lower intrinsic D2 receptor activity is associated with a lower risk of akathisia, activation, and insomnia, which was particularly relevant given the patient’s preexisting hyperactivity and irritability. In addition, brexpiprazole has been associated with relatively lower metabolic liability than several other antipsychotics, and tolerability was favorable in the present case [15].

Brexpiprazole also exhibits relatively strong partial agonistic activity at 5-HT1A receptors, which may contribute to its anxiolytic effects [8]. In the present case, a reduction in anxiety may have contributed to reduced resistance to discarding possessions.

In the present case, brexpiprazole treatment may have been associated with modest improvements in hoarding behaviors, functional outcomes, and patient autonomy. However, this study is limited by its nature as a single case report and the relatively short follow-up period. In addition, improvements were mainly observed in discarding symptoms and some functional domains, whereas persistent aggression and difficulty accepting guidance showed little change. Therefore, the treatment response should be interpreted cautiously. Continued multidisciplinary support aimed at stabilizing the patient’s environment and maintaining safety was considered essential, with priority placed on preserving the therapeutic relationship rather than rapidly advancing psychological or behavioral interventions. Ongoing support for both parents may also have contributed to treatment adherence.

Although symptom improvement was limited, the present case highlights the potential importance of reducing anxiety and emotional dysregulation in complex neurodevelopmental conditions. Severe aggression, irritability, and emotional outbursts remained major clinical challenges, underscoring the need for further pharmacological and psychotherapeutic approaches targeting emotional dysregulation. Further research is needed to clarify optimal treatment strategies for complex cases involving ASD, ADHD, multiple psychiatric comorbidities, and severe disruptive behaviors.

## Conclusions

This case highlights the clinical complexity and treatment resistance often observed in children with comorbid ASD and ADHD accompanied by multiple psychiatric conditions. The findings suggest that cumulative traumatic experiences, together with heightened anxiety and emotional dysregulation, may contribute to the development and persistence of HD. Although brexpiprazole treatment was associated with partial improvement in hoarding behaviors and separation anxiety, its long-term clinical benefit remains uncertain and should be interpreted cautiously. Further accumulation of well-documented case reports is needed to refine therapeutic strategies and improve clinical outcomes in this challenging population.
